# Hematological Effects of Gold Nanorods on Erythrocytes: Hemolysis and Hemoglobin Conformational and Functional Changes

**DOI:** 10.1002/advs.201700296

**Published:** 2017-09-25

**Authors:** Xingchen Zhao, Dawei Lu, Qian S. Liu, Yiling Li, Rui Feng, Fang Hao, Guangbo Qu, Qunfang Zhou, Guibin Jiang

**Affiliations:** ^1^ State Key Laboratory of Environmental Chemistry and Ecotoxicology Research Center for Eco‐Environmental Sciences Chinese Academy of Sciences Beijing 100085 P. R.China; ^2^ College of Resources and Environment University of Chinese Academy of Sciences Beijing 100049 P. R. China

**Keywords:** bioconjugation, erythrocyte, gold nanorods, hemoglobin, hemolysis

## Abstract

Gold nanorods (GNRs) are a unique class of metal nanostructures that have attractive potentials in biomedical applications, and the concern on their biological safety is concomitantly increasing. Hemocompatibility is extremely important as their contact with blood circulation is unavoidable during in vivo delivery. Herein, two kinds of GNRs coated with hexadecyltrimethylammonium bromide (C‐GNRs) or poly(sodium‐*p*‐styrenesulfonate) are used to test their potential toxicological effects in blood. C‐GNRs with positive surface charges efficiently induce hemolysis when encountering erythrocytes. Cellular internalization of C‐GNRs is found, and they subsequently bind with hemoglobin, forming bioconjugates. The interaction between hemoglobin and C‐GNR (stoichiometry 32.7:1) is regulated by electrostatic forces. Chromophores like tryptophan (Trp) are found to interact with C‐GNRs, causing enhancement in fluorescence intensity. The conformation of protein is partially altered, evidenced by decrease in α‐helical, increase in β‐sheet and random coil of hemoglobin. Although C‐GNRs do not essentially decrease oxygen binding capacity of hemoglobin, they hamper oxygen release from the protein. Heme, the oxygen binding unit, releases from hemoglobin upon C‐GNR treatment, which could contribute to C‐GNR‐induced hemolysis. This study demonstrates the hematological effects of GNRs, revealing their potential risk in biomedical applications.

## 1 Introduction

Gold nanorods (GNRs) possess promising application perspectives in many fields, like drug delivery vehicle, biomedical imaging, and photothermal therapy, due to their excellent physiochemical and optical properties.^1^ Although the development of GNRs is rapidly increasing, their toxicity evaluation is imperative, regarding the biosafety issue in diverse applications, especially in medical areas.^2^ Despite the previously reported diverse cytotoxicities,^3^ the hemocompatibility evaluation and improvement of the nanomaterial is of great importance, considering that most of the GNRs are delivered to the target organs through blood circulation system; and special attention should be paid to the possible hematological effects of GNRs when they encounter erythrocytes in blood.

Erythrocytes, which are dominant cells in blood (99%), play a vital role in carrying oxygen from the lungs to tissues or organs to meet metabolic needs in vivo.^4^ However, they are very vulnerable to exogenous toxicants, showing diverse toxicological effects such as changes in membrane protein contents, prolonged hemolysis, and methemoglobinemia.^5^ As for nanotoxicological aspects, fine particles and nanoparticles were visualized to penetrate the red blood cell membrane via an unknown mechanism different from phagocytosis and endocytosis.^6^ The abnormal sedimentation, hemagglutination, and dose‐dependent hemolysis of erythrocytes were induced by titanium dioxide nanoparticles (nano‐TiO_2_), which was involved with changes in surface native properties, nano‐TiO_2_ *trans*‐membrane, and oxidative stress.^7^ More erythrocyte toxicities, such as deformation, agglutination, and membrane damage, were extensively reported for some other nanomaterials.^8^ Although biologically incompatible responses of gold nanoparticles in blood have been noticed before,^9^ the interaction between GNRs and erythrocytes still needs more investigations. Hemoglobin, as the most abundant protein in erythrocytes, mediates the main physiological functions in erythrocytes. The interferences of toxicants to hemoglobin are crucial for the evaluation of their hemocompatibility.^10^ When entering erythrocytes, how GNRs react with hemoglobin would be of high importance in explaining the related toxicological effects.

In this study, we compared the hemolytic effects of two kinds of GNRs, including hexadecyltrimethylammonium bromide (CTAB)‐coated GNRs (C‐GNR) and poly(sodium‐*p*‐styrenesulfonate) (PSS)‐coated GNRs (P‐GNRs) at concentrations comparable to those used in previous biomedical studies.^11^ The interaction between C‐GNRs and hemoglobin were further studied by employing scanning transmission electron microscopy (STEM), dynamic light scattering (DLS), and zeta‐potential analysis. Alterations in protein structure, microenvironment of amino acids, and protein function were determined by photometric analysis upon the treatment of C‐GNRs. The thermodynamic parameters, including association constant, binding number, Δ*H*°, Δ*S*°, and Δ*G*° of hemoglobin binding to C‐GNRs measured by isothermal titration calorimetry (ITC) revealed the binding mechanism. The functional change in hemoglobin and its heme release were further discussed. These findings may offer salient clues on potential health risks from C‐GNRs due to their medical use or environmental exposure.

## 2 Results and Discussion

### 2.1 Erythrocyte Hemolysis Induced by C‐GNRs

**Figure 1:**
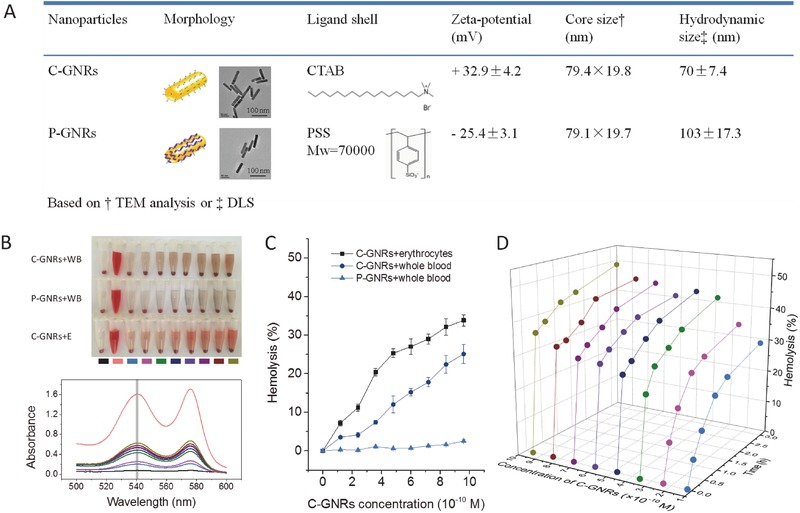
A) Physical and chemical properties of GNRs coated with CTAB and PSS, respectively. B) Photographs of the erythrocytes treated by two kinds of GNRs (upper panel), and the absorption spectra of the hemolytic supernatant samples from the group of C‐GNRs+E (bottom panel). WB is whole blood and E is erythrocyte dispersion. The test groups included PBS (black, negative control), water (pink, positive control), 1.2 × 10^−10^ (blue), 2.4 × 10^−10^ (magenta), 3.6 × 10^−10^ (olive), 4.8 × 10^−10^ (navy), 6 × 10^−10^ (purple), 7.2 × 10^−10^ (violet), 8.4 × 10^−10^ (wine), and 9.6 × 10^−10^ m (dark yellow) GNRs, respectively. The incubation time was 0.2 h. C) The quantitative analysis based on λ_541 nm_ for the concentration‐dependent hemolysis induced by two kinds of GNRs in whole blood and erythrocyte dispersion. D) Time‐course for hemolysis upon the stimulation of C‐GNRs at different concentrations in erythrocyte dispersion.

Two types of GNRs, coated with PSS or CTAB, were selected in this study to compare the influences of surface charge on potential erythrocytic effects. Their physical and chemical properties are given in **Figure** **1**A and Figure S1 (Supporting Information), showing they possess very similar characteristics, including morphology (evenly distributed rod‐shaped), core size (79 nm), aquatic dispersion (light brown), and the extinction spectrum (λ_max_ ≈520 and 800 nm), but distinct surface charges (32.9 mV for C‐GNRs, −25.4 mV for P‐GNRs) due to different coating molecules.

Using mouse whole blood (WB), the potential hematological effects of GNRs were investigated, and the photographic results showed that C‐GNRs significantly induced hemolysis in erythrocytes, while no obvious response was observed upon P‐GNR stimulation at the same concentration range (Figure 1B, upper panel). Similarly, when the erythrocyte dispersion in phosphate buffered saline (PBS) was analyzed, C‐GNRs caused more severe hemolysis, when compared to the whole blood test (Figure 1B, upper panel). The characteristic absorbance spectrum was scanned for hemoglobin released from erythrocytes upon the treatments with different concentrations of C‐GNRs (Figure 1B, bottom panel). Based on the absorbance at 541 nm, the hemolysis ratio was quantitatively calculated and the results showed that the positively charged nanoparticles exerted higher hemolytic activity than the negatively charged ones, and the pure erythrocytes in PBS were more vulnerable to nanorod stimulation than those in whole blood (Figure 1C), which was similar to the previous finding.^12^ Additionally, hemolysis of erythrocytes induced by C‐GNR was instant (within 0.2 h), nevertheless exhibited in time‐dependent and concentration‐related manners (Figure 1D and Figure S2, Supporting Information).

The surface chemistry dependent effects have been discussed for GNRs on their uptake, toxicity, and gene expression.^13^ GNRs stabilized with CTAB were found to cause more severe cellular responses than those coated with either thiolated polyethylene glycol 5000 or mercaptohexadecanoic acid.^14^ Consistent with previous findings that the cytotoxicity of nanoparticles was strongly correlated to their surface charges, the toxicity disparity stemmed from different electrostatic associations between nanoparticles and cell membranes and the membrane‐compromising effects of CTAB.^9^, ^10^, ^13^ C‐GNRs with positive surface charges would electrostatically attach to negatively charged erythrocytes, where (1) binding of CTAB surface of GNRs with the phosphatidyl choline‐rich erythrocyte membrane and (2) bending of the erythrocyte membrane to adapt to the rigid surface of GNRs might occur.^15^ Lau et al. found that the toxicity of C‐GNRs leading to hemolysis through the Ca^2+^ pathway was largely due to the unbound CTAB residues or CTAB bilayer of C‐GNRs.^12^ Hemolytic effects of CTAB at equal amounts to those loaded onto C‐GNRs were thus evaluated (Figure S3, Supporting Information), and the results demonstrated that CTAB caused slight hemolysis in either whole blood or erythrocyte suspension, which was less severe than the condition in C‐GNR exposure groups (Figure 1C). This emphasizes the particulate effect of C‐GNRs on erythrocytic hemolysis other than the surface coating of CTAB. C‐GNRs potentially provided large cell‐contactable surfaces, recruiting a locally high concentration of CTAB for the reaction with erythrocytes.

### 2.2 Cellular Internalization of C‐GNRs in Erythrocytes and Identification of the Main Binding Protein

**Figure 2:**
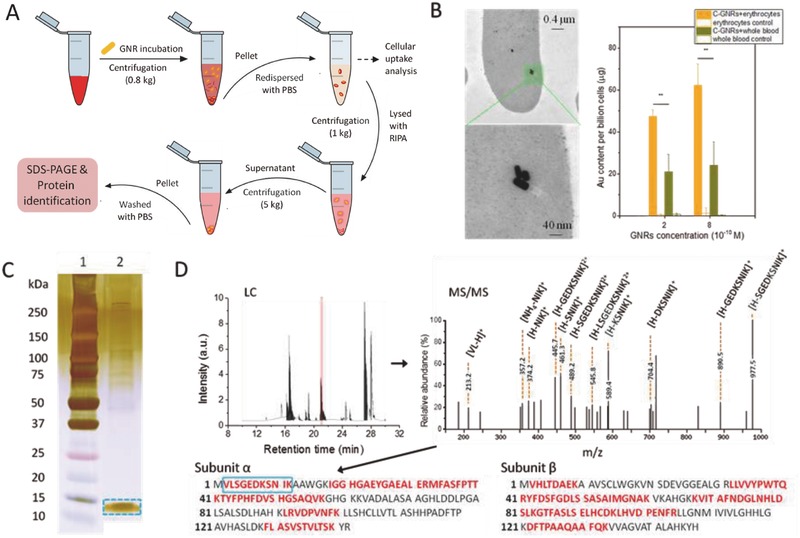
A) Scheme of the experimental procedure for cellular internalization of C‐GNRs and the identification of their binding protein. B) TEM images for the cellular internalization of C‐GNRs (left panel), and the quantitative results of Au levels in erythrocytes (right panel). C) SDS‐PAGE analysis of the erythrocyte samples incubated with 9.6 × 10^−10^ m C‐GNRs for 0.2 h. D) The LC spectrum of the trypsin‐digested peptide samples from SDS‐PAGE (left panel), and the representative MS/MS data for LC peak with retention time of 21.1 min, which was characterized as the sequence of VLSGEDKSNIK (right panel). Based on protein database matching, the peptide sequences marked in red were identified, which covered 50% of α subunit and 59% of β subunit sequences of hemoglobin.

Besides the significant hemolytic effects of C‐GNRs on erythrocytes, their cellular internalization was characterized by transmission electronic microscopy (TEM) and inductively coupled plasma mass spectrometry (ICP‐MS). The whole blood samples incubated with C‐GNRs were processed as indicated in **Figure** **2**A, and the results confirmed the existence of C‐GNRs in cells, which showed encapsulation of C‐GNRs in the cells. Likewise, cellular penetration and encapsulation was previously reported for spherical positively charged gold nanoparticles in erythrocytes,^6^ where the mechanism was different from phagocytosis and endocytosis. Au levels were increased in a concentration‐dependent manner in erythrocytes upon C‐GNR treatment (Figure 2B). The cellular internalization of C‐GNRs in a pure erythrocyte system was more efficient than that in whole blood, which was consistent with the hemolytic effect observed earlier.

To further clarify how C‐GNRs interacted with erythrocytes, the major binding protein of cellular internalized C‐GNRs was analyzed. As prepared by the protocol depicted in Figure 2A, the extracted GNR‐binding proteins were eventually submitted to sodium dodecyl sulfate‐polyacrylamide gel electrophoresis (SDS‐PAGE) and subsequent liquid chromatography‐tandem mass spectrometry (LC‐MS/MS) analysis. The molecular weight of the dominant protein band in Lane 2 (GNR‐binding protein sample) was around ≈15 kDa (Figure 2C), which was close to that of hemoglobin subunit. LC‐MS/MS analysis of the trypsin‐digested samples from this dominant band further confirmed the existence of the peptides marked in red, which covered 50% of α subunit and 59% of β subunit sequences of hemoglobin. As indicated in Figure 2D, LC peak with the retention time of 21.1 min was demonstrated as an example. Its MS data showed the characteristic fragments carrying one or two charges with the addition of H^+^ or NH_4_^+^ after electrospray ionization (e.g., [VL‐H]^+^ ionized on N terminal fragment and [NH_4_‐NIK]^+^ ionized on C terminal fragment). The search for sequence homology in nonredundant protein database proved the peptide was VLSGEDKSNIK, which belonged to subunit α of hemoglobin. Consequently, the results demonstrated here provided the substantial evidence that the major constituent of protein corona bound with C‐GNRs was hemoglobin in erythrocytes.

### 2.3 Characterization of C‐GNRs Bioconjugated with Hemoglobin

**Figure 3:**
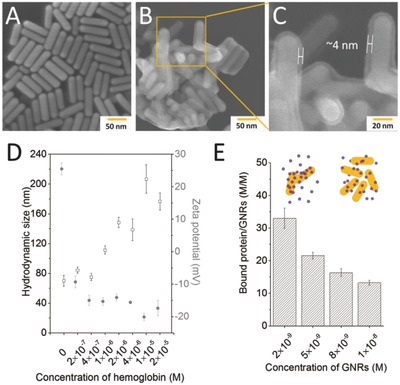
STEM images of A) native and B,C) hemoglobin‐bioconjugated C‐GNRs. D) Hydrodynamic size and zeta‐potential changes of C‐GNRs (4 × 10^−10^ m) caused by the addition of hemoglobin at different concentrations (*n* = 3). E) Hemoglobin adsorption efficiencies on C‐GNRs at different concentrations. The concentration of hemoglobin was controlled at 5 × 10^−7^ m, *n* = 3.

The interaction of C‐GNRs with hemoglobin was further characterized by STEM. As indicated in **Figure** **3**A, native C‐GNRs were evenly distributed with clear borders. Comparatively, C‐GNRs bioconjugated with hemoglobin exhibited blur edges (Figure 3B), showing protein attachment on C‐GNR surface. Obvious aggregates of hemoglobin‐bound C‐GNRs were formed, which was distinct from the good dispersion of native C‐GNRs. The partial magnification image of Figure 3C clearly revealed C‐GNRs were covered uniformly by protein corona layer with a thickness of about 4 nm. Therefore, a monolayer of hemoglobin was probably bioconjugated as this protein was reported to have a quaternary structure characteristic of many multisubunit globular proteins, and the diameter of a single hemoglobin molecule was about 5 nm.^16^ The obvious difference between native C‐GNRs and the bioconjugates originating from a composite protein layer was extensively observed in the scans from STEM analysis, showing the universal protein binding on C‐GNRs after hemoglobin incubation.

Dynamic light scattering determines the size distribution profile of small particles in suspension. To further investigate the interactions between C‐GNRs and hemoglobin, the hydrodynamic particle size of pure C‐GNRs was compared with that of the bioconjugates. The result in Figure 3D shows that C‐GNRs are near‐monodispersed with a mean hydrodynamic diameter of 70 nm. With the increasing bioconjugation of hemoglobin, the intensity‐weighted average particle diameters increased, finally reaching a platform of about 180 nm. The significant increase in hydrodynamic size of hemoglobin‐bioconjugated C‐GNRs (110 nm) could be equal to at least 20 layers of hemoglobin bioconjugated on the particles in thickness if there was no aggregation. However, as indicated by STEM, hemoglobin was bioconjugated on the surface of C‐GNRs and formed a layer of about 4 nm, which meant that the increment in hydrodynamic size might be attributed to the formation of nanoparticle aggregates. Protein‐induced nanomaterial aggregation was also reported in our previous study, wherein electrostatic attraction triggered the agglomeration of serum albumin molecules and carbon nanotubes.^17^ However, due to the effect of “dynamic adsorption balance,”^18^ excess protein would not cause the unlimited increase in nanomaterial aggregation. That was why the hydrated particle size of C‐GNR bioconjugates became stable at around 180 nm when the protein concentration was increased to 4 × 10^−5^ m.

Zeta potential measurement tells one of the important characters of nanomaterial surface coating, which is commonly used to characterize the changes of the nanoparticles due to their potential interactions with some other materials. Herein, zeta potentials of C‐GNRs were tested in the absence or presence of hemoglobin at different concentrations. The results in Figure 3D demonstrated that naked C‐GNRs were positively charged with zeta potential of 25.5 mV. The addition of hemoglobin caused rapid decrease in zeta potentials of the bioconjugates and the lowest value was −17.4 mV when the so‐called “dynamic adsorption balance” was reached.^19^ Due to electrostatic attraction, the negatively charged protein easily absorbed onto the positively charged surface of nanoparticles, thus causing dramatic drop of zeta potential by 42.9 mV when 2 × 10^−5^ m hemoglobin was introduced into C‐GNR suspensions. The alterations in zeta potentials were consistent with the changes of hydrated particle sizes along with the increasing concentrations of hemoglobin in the incubation system.

The bioconjugation of hemoglobin with C‐GNRs were further estimated by the amounts of residue protein in the incubation systems using SDS‐PAGE and bicinchoninic acid (BCA) assays. The result in Figure S4 (Supporting Information) shows that the increase of C‐GNR concentration in the incubation system causes the decrease of free hemoglobin content in the supernatant, as evidenced by fading band intensity in SDS‐PAGE. On the flip side, the amount of bioconjugated hemoglobin on C‐GNRs apparently increased. The bioconjugation ratios of hemoglobin on C‐GNRs were quantitatively evaluated by the supernatant free hemoglobin concentrations based on BCA assay. As shown in Figure 3E, the protein adsorption efficiency was negatively correlated with C‐GNR concentration. It was reasonable the protein amount exposed to each particle would be reduced when a fixed amount of hemoglobin was mixed with increasing concentrations of C‐GNRs. Meanwhile, the protein‐nanoparticle dynamic adsorption balance guaranteed that C‐GNRs at high concentrations could not occupy more protein even though there was still unbound protein available in solution.

### 2.4 Conformational Changes of Hemoglobin upon C‐GNR Bioconjugation

**Figure 4:**
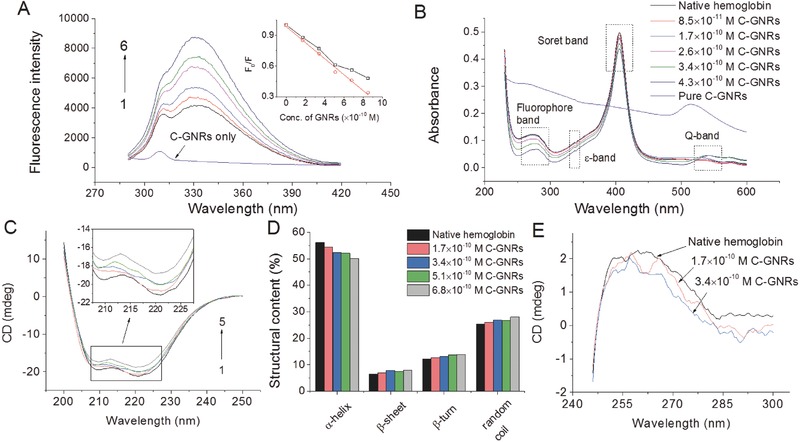
Conformational changes of hemoglobin upon C‐GNR bioconjugation. A) Fluorescence spectra of hemoglobin in absence and presence of C‐GNRs. The inset is the raw data with inner filter effect (black) and the corresponding Stern–Volmer plot (red). *C*_hemoglobin_ = 1 × 10^−6^ m and *C*_GNRs_ (from 1 to 6) = 0, 1.7, 3.4, 5.1, 6.8, and 8.5 × 10^−10^ m, respectively. Spectrum of C‐GNRs (8.5 × 10^−10^ m) was also recorded. B) UV–vis absorbance spectra of hemoglobin incubated with different concentrations of C‐GNRs. Final concentration of hemoglobin is 5 × 10^−7^ m, and pure C‐GNRs (4.3 × 10^−10^ m) is tested as the control. C) The CD spectra of hemoglobin bioconjugated with different concentrations of C‐GNRs. The concentration of hemoglobin was controlled at 2 × 10^−6^ m, and the concentrations of C‐GNRs in (C) marked from 1 to 5 were 0, 1.7, 3.4, 5.1, and 6.8 × 10^−10^ m, respectively. D) The structural contents for the secondary parameters of hemoglobin bioconjugated with different amounts of C‐GNRs. E) Near‐UV CD spectra of hemoglobin bioconjugated with C‐GNRs. Incubation time in these tests was 0.5 h.

The fluorescent intensity of hemoglobin coupling to the longitudinal plasmon resonance could be increased due to the longitudinal field enhancement of GNRs.^20^ The theory of longitudinal field was well developed and believed to be effective in describing the linear or nonlinear optical relationships from rough metal surface.^21^ GNRs are known to enhance the fluorescence emission intensity and decrease the molecular excited‐state lifetimes of interacting fluorophores.^22^ The fluorescence enhancement is attributable to a combination of processes including enhanced absorption and modification of the decay rate of the molecule, and enhanced coupling efficiency of the fluorescent emission to the far field.^23^ As shown in **Figure** **4**A, the fluorescence intensity of hemoglobin increased with the addition of C‐GNRs, which was different from the previous findings that the protein fluorescence quenching occurred with the binding of nanomaterials.^24^, ^25^ When the fluorescence intensity at the maximum emission wavelength (334 nm) was evaluated, it was found that *F*_0_/*F* value (the ratio between the protein fluorescence intensity without and with the addition of C‐GNRs) was negatively linearly related with the incubation concentrations of C‐GNRs. The increase in protein fluorescence intensity could be attributed to the fact that hemoglobin got unfolded upon the interaction with C‐GNRs, and more fluorophores tryptophan (Trp), phenylalanine (Phe), and tyrosine (Tyr) in the protein were exposed, thus inducing locally increased index of refraction. The closer the exposed fluorophores reached the GNR surface, the stronger were surface plasmon resonance effects induced.^26^ The fluorescence intensity of fluorophores could be significantly enhanced when these molecules were in close proximity to C‐GNRs at increasing levels.

The UV–vis absorption spectra of hemoglobin and its C‐GNR bioconjugates give information on detailed structural changes. According to the previous study,^27^ the UV–vis spectra of pristine hemoglobin exhibit several characteristic electronic bands located at ≈280 nm (corresponding to phenyl group of Trp and Tyr residues), ≈350 nm (ε band), ≈405 nm (Soret or heme band, π→π* electronic transition of heme structure), ≈540 nm, and ≈575 nm (oxy‐band or Q‐band). The protein profiles of fluorophore band, ε band, Soret band, as well as Q‐band might be altered by interacting with C‐GNRs. Figure 4B shows the typical UV–vis absorbance spectrum of hemoglobin (black line). The strong Soret band at 406 nm was from heme group burying in a hydrophobic cavity formed by the backbone of hemoglobin through appropriate folding. Adsorption of Soret band assigned to the porphyrin ring was expected to provide insightful information on the possible unfolding or denaturation of hemoglobin during conjugation. Alterations in the absorbance at Soret band indicated structural changes in native protein form. As shown in Figure 4B, the addition of C‐GNRs caused a concentration‐related hypochromism of Soret band without red or blueshifts, mainly due to the nonexposure of heme system from the crevices to the exterior part of the subunit.^28^

Q‐band of hemoglobin is associated with the microenvironment of heme group. In contrast to Soret band, the absorbance of Q‐band increased with the gradual addition of C‐GNRs (Figure 4B). What's more, Q‐band of native hemoglobin showed a maximum absorbance at 535 nm, and it was redshifted to 543 nm after the addition of C‐GNRs, demonstrating different microenvironment surroundings of the heme after bioconjugation with C‐GNRs. These spectrometric phenomena documented that heme group experienced some potential perturbation due to the interaction of hemoglobin with C‐GNRs through electrostatic force.^29^ The time‐dependent conformational alterations of hemoglobin were also studied by incubating hemoglobin with GNRs for 0.5, 1, 2, 4, and 8 h, respectively. As shown in Figure S5 (Supporting Information), all the characteristic peaks remained unchanged in both intensities and positions as the incubation time was extended to 8 h, indicating that C‐GNRs induced an instant conformational change in hemoglobin within 0.5 h. This phenomenon was different from previous findings that graphene oxide‐induced catalase structural changes happened slowly as incubation time was extended.^30^

Alteration in the secondary structure of hemoglobin upon the bioconjugation with C‐GNRs was evaluated by circular dichroism (CD) analysis. The far ultraviolet CD spectrum of hemoglobin showed two negative peaks at 208 and 222 nm, which were due to *n*→π* and π→π* transition of α‐helix, respectively. As shown in Figure 4C, there was a significant increase of hemoglobin CD ellipticity upon the addition of C‐GNRs without imposing any shift of peak positions. This clearly indicated that the binding of hemoglobin to C‐GNRs induced some modifications in the secondary structural content of the protein.^31^ According to the calculation of the secondary structure parameters, it was found that hemoglobin itself contained around 56.1% α‐helix (Figure 4D). Upon the interaction with C‐GNRs, the percentages of α‐helix gradually decreased to 50.1%, suggesting the disruption of H‐bonding and the decrease in the helical content of hemoglobin. Meanwhile, β‐sheet content increased from 6.4% to 8%, which was associated with the increment of random coil content. Therefore, the binding of C‐GNRs induced unfolding of hemoglobin and the loss of a large part of helical stability, resulting in polypeptide chain extension.^17^, ^32^ Based on the findings described earlier, it could be deduced that the binding of C‐GNRs caused extensive conformational changes in hemoglobin, and the protein adapted its structural content during the interaction process.

Near‐UV CD spectra (250–300 nm) provide useful information on the tertiary structure of hemoglobin. Chromophores such as Phe (262–267 nm) along with Tyr and Trp (282–290 nm) exhibit fine characteristic peaks in this region. Alterations of the signal intensities in near‐UV CD spectra reveal structural changes of hemoglobin as well. As shown in Figure 4E, decrement in CD intensity clearly indicated the perturbation of C‐GNRs on 3D configuration of hemoglobin, leading to unfolding of the native protein structure. This phenomenon was in line with redshift in fluorescence measurements of hemoglobin upon C‐GNR bioconjugation (Figure 4B).

### 2.5 Thermodynamic Nature of C‐GNR Bioconjugation with Hemoglobin

**Figure 5:**
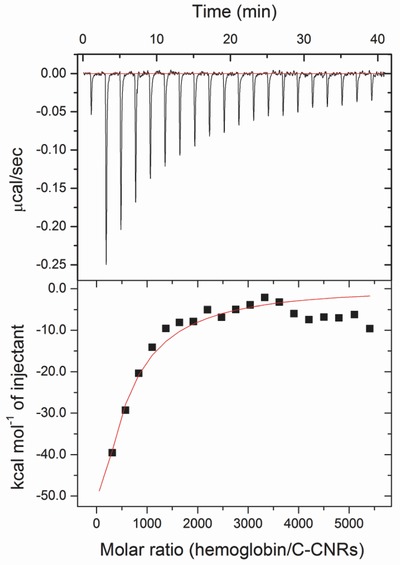
ITC profile of C‐GNR interaction with hemoglobin at 298 K. The calorimetric responses were real‐time monitored for the titration of C‐GNRs with successive injections of hemoglobin at pH 7.5.

Analysis of calorimetric curve was performed using nonlinear fitting of Microcal Origin (**Figure** **5**). The best fitting for integrated heats was obtained using a single site of the binding model, where the binding affinity (*K*_A_), the binding stoichiometry (*n*), and the binding thermodynamics could be calculated. The Gibbs free energy Δ*G*° and the entropy Δ*S*° of the binding reaction could be obtained by Δ*G* = Δ*H* −*T*Δ*S* = −*RT*In*K*.^24^ All binding parameters are shown in **Table** **1**. The negative Δ*G*° indicated that the binding was an exothermic process. The calculated affinity constant of *K*_A_ was 1.05 × 10^6^ m ^−1^, manifesting the high affinity occurred between C‐GNRs and hemoglobin. Besides, for each C‐GNR, about 33 protein molecules could adsorb onto its surface when the binding event reached saturation. Since both the enthalpy and the entropy of the binding were negative, the binding was entropically opposed but enthalpically favored at the experimental temperature.

**Table 1:** ITC thermodynamic parameters for the interaction between hemoglobin and C‐GNRs at 298 K

*K*_A_ [M^−1^]	*n*	Δ*G*° [J mol^−1^]	Δ*S*° [J mol^−1^ K^−1^]	Δ*H*° [J mol^−1^]
1.05 × 10^6^	32.7	−3.58 × 10^4^	−2.79 × 10^4^	−8.35 × 10^6^

The binding of biomacromolecules to nanoparticles such as GNRs might involve the formation of weak noncovalent forces including hydrogen bonds, van der Waals forces, electrostatic forces, and hydrophobic interaction forces.^33^ Elucidating the thermodynamic parameters might help provide clues on the involvement of these forces in the conjugation process. According to the view of Ross,^34^ when Δ*H*° < 0 and Δ*S*° > 0, the main force was due to electrostatic interactions; Δ*H*° < 0 and Δ*S*° < 0 were associated with hydrogen bonding or van der Waals forces, and Δ*H*° > 0 and Δ*S*° > 0 resulted in hydrophobic interactions. The negative values obtained for both Δ*H*° (−8.35 × 10^6^ J mol^−1^) and Δ*S*° (−2.79 × 10^4^ J mol^−1^ K^−1^) in this study suggested the involvement of electrostatic interaction in the formation of C‐GNR–hemoglobin bioconjugates. As revealed earlier, GNRs coated with CTAB were positively charged on the surfaces. Hemoglobin was a negatively charged molecule at pH 7.5 with pI of 6.8, thus it was expected to bind with C‐GNRs at the sites consisting of negatively charged amino acid residues.

### 2.6 Functional Change and Heme Release of Hemoglobin upon C‐GNR Bioconjugation

**Figure 6:**
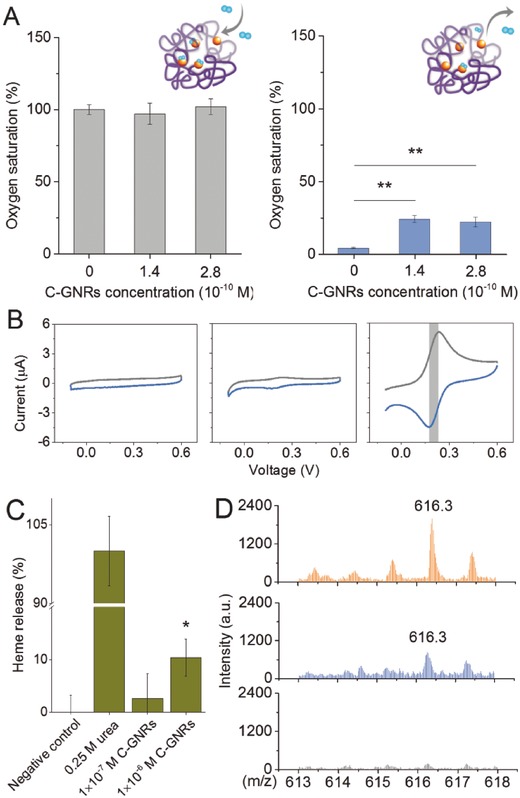
A) Oxygen binding (left) and releasing (right) capacities of hemoglobin (1.4 × 10^−6^ m) incubated with different concentrations of C‐GNRs. B) The cyclic voltammetry curves of C‐GNRs (left panel), hemoglobin (middle panel), and C‐GNRs bioconjugated with hemoglobin (right panel). C) The bioconjugation of C‐GNRs caused heme release from hemoglobin. D) MALDI‐TOF‐MS analysis of heme release from hemoglobin (4 × 10^−4^ m). Upper, middle, and lower panels represent hemoglobin samples treated with 0.25 m urea (positive control), 1 × 10^−6^ m C‐GNRs, and PBS (negative control), respectively. *p < 0.05 versus negative control.

The main function of hemoglobin is to carry oxygen from the lungs to the other part of the body, where it releases oxygen to permit aerobic respiration of tissue cells. Therefore, the functional changes of hemoglobin were evaluated by testing its oxygen binding and releasing abilities upon C‐GNR treatments. Based on spectrophotometric absorption differences in hemoglobin and oxyhemoglobin, it was found that the bioconjugation of hemoglobin with C‐GNRs did not influence oxygen saturation in oxygen‐rich condition (**Figure** **6**A, left panel), showing oxygen binding ability of hemoglobin was unchanged. However, under oxygen‐deficient mode, oxygen saturation in C‐GNR exposure groups (22–24%) was significantly higher than that in the control (4%) (Figure 6A, right), showing the bioconjugation of C‐GNRs with hemoglobin impeded the release of oxygen from the protein. Clinically, the functional change of hemoglobin could be potentially related with hypoxia, which might cause severe deleterious effects on crucial organs such as the brain.

pH was found to regulate oxygen binding of hemoglobin. The oxygen binding ability could partially recover in the presence of H^+^ (Figure S7, Supporting Information). Take β subunit as an example, as pH decreased, the side chain of histidine β146 was protonated, the salt bridge with aspartate β94 was formed, and the quaternary structure characteristic of deoxyhemoglobin was stabilized, leading to a greater tendency for oxygen to be released. When compared to the negative control (NC) of pure protein alone, the oxygen saturation ratio in protein samples treated with C‐GNRs was relatively higher, showing incomplete O_2_ release from hemoglobin (Figure S7, Supporting Information).

In quest of the underlying mechanism how C‐GNRs hampered oxygen release from hemoglobin, the electron transfer characteristics of the protein assembled on C‐GNRs were investigated. The cyclic voltammetry (CV) responses showed a pair of redox peaks with a formal potential (*E*_0_) of about 0.205 V in the group of C‐GNRs bioconjugated with hemoglobin (Figure 6B), when compared to no obvious signals in the groups of C‐GNRs and hemoglobin alone. It was thus assumed that when heme group approached C‐GNRs, the electrons were transferred efficiently from iron atom (Fe^2+^) of heme to C‐GNRs and then to the electrode. For native hemoglobin, heme structure was deeply buried in the hydrophobic cavity of hemoglobin, where there was a long distance from the electrode and hence poor electrochemical signal was detected due to large steric hindrance. A conformational change in histidine residue (His‐α58 and His‐β63) toward porphyrin in hemoglobin molecule structure was essential in oxygen release. In this process, the protein molecule adjusted its own quaternary structure and “pulled” iron atom through histidine residue (Figure S6, Supporting Information), where oxygen molecule was released because of steric hindrance (N→Fe coordination was much stronger than that of O→Fe).^35^ As mentioned earlier, the binding of hemoglobin to C‐GNRs induced conformational changes in the protein, especially the microenvironment around histidine and heme, which would subsequently make them incapable of releasing O_2_.

Heme release could cause dysfunction of hemoglobin, which contributed to subsequent hemolysis of erythrocytes.^36^ Based on spectroscopic analysis of heme extract from different groups, it was indicated that hemoglobin incubation with C‐GNRs caused concentration‐dependent heme release (Figure 6C). Further experiment using matrix‐assisted laser desorption/ionization time of flight mass spectrometry (MALDI‐TOF‐MS) analysis gave substantial evidence that the abundance of heme fragments (*m/z* = 616.3) was obviously enhanced in hemoglobin treated with C‐GNRs compared to free protein (Figure 6D). Without being bound to proximal histidine (His‐α58 and His‐β63) at the fifth coordination position of Fe, free heme monomer was unable to release oxygen itself, which explained the relatively higher oxygen saturation in oxygen deficient condition (Figure 6A, right panel). The lipophilic molecule, heme, which was recognized as a hemolytic agent, could intercalate in the membrane and impair the lipid bilayers in erythrocytes.^37^ The increase in heme release induced by C‐GNRs could be responsible for the hemolysis observed in Figure 1. Considering the potential effect from surface coating molecular, CTAB at the concentrations equal to those coated on GNRs was also tested for its effect on heme release (Figure S8, Supporting Information), and the results indicated that heme release did happen, but was much lower than that in C‐GNR groups, showing particulate‐specific effect of C‐GNRs.

## 3 Conclusions

The potential hematological effects were studied for two kinds of GNRs, and C‐GNRs were found to cause efficient hemolysis of erythrocytes. After their intracellular internalization, C‐GNRs were predominantly bound with hemoglobin. The instant molecular interaction between hemoglobin and C‐GNRs resulted in the fluorescence enhancement wherein Trp residues interacted with positively charged GNRs surfaces. C‐GNRs caused the perturbation around heme group, generating conformational heterogeneity in hemoglobin‐GNRs bioconjugates. The bound hemoglobin partially lost α‐helical content and gained β‐sheet and random coil structures. One C‐GNR was able to bind with 33 protein molecules through electrostatic attractions. Moreover, structural changes of hemoglobin induced by C‐GNRs substantially caused decreased oxygen release and increased heme release, resulting in functional changes of the protein and hemolysis of erythrocytes. The findings on blood biocompatibility of C‐GNRs have provided useful information on in vivo risk assessment of this kind of nanomaterials from biomedical and toxicological aspects.

## 4 Experimental Section

*Materials*: Human hemoglobin powder, purchased from Sigma‐Aldrich (Missouri, USA), was reconstituted in ultrapure water (Millipore, Billerica, USA) to form stock solution (1 × 10^−5^ m, in 0.2 m PBS, pH 7.5), and was stored at −80 °C. Unless stated otherwise, the other chemicals bought from Sinopharm Co., Ltd. (Beijing, China) were of analytical grade and kept in darkness at 4 °C.

*Preparation of GNRs Coated with CTAB or PSS*: C‐GNRs were prepared using the method from Ye et al.^38^ Briefly, CTAB (7.0 g) and NaOL (1.234 g) were dissolved in warm water (250 mL, 50 °C), after which AgNO_3_ (18 mL, 4 × 10^−3^ m) and HAuCl_4_ (250 mL, 1 × 10^−3^ m) solutions were introduced. The solution became colorless after 90 min stirring, and HCl (2.1 mL, 36.5%) was subsequently added. After another 15 min slow stirring at 400 rpm, ascorbic acid (1.25 mL, 0.064 m) was added. After stirring violently for 30 s, the growth solution was ready. The seed solution was prepared by adding NaBH_4_ (1 mL, 6 × 10^−3^ m) to HAuCl_4_ (10 mL, 0.25 × 10^−3^ m) solution containing CTAB (0.1 × 10^−3^ m) under vigorous stirring for 2 min, and then it was aged for 30 min. The seed solution (0.8 mL) was injected into the growth solution, and the resultant mixture was stirred for 30 s. Finally, it was left undisturbed overnight at 30 °C for C‐GNR growth. C‐GNR pellets were collected after three times wash (H_2_O) and centrifugation (5000 g, 15 min). Modification of GNRs with PSS was performed for the preparation of P‐GNRs according to the protocol from Leonov et al.^39^ The stock solution of GNRs (2.6 × 10^−5^ m) was prepared by dispersing the nanorods in distilled water.

*Characterization of GNRs*: Both GNRs were characterized for their morphology, zeta potentials, particle lengths (core size), and hydrodynamic sizes. GNR dispersion (200 µL) was loaded onto carbon film coated TEM copper grids, and air‐dried overnight. The as‐prepared samples were submitted to the observation by a Hitachi S‐5500 field‐emission STEM (Tokyo, Japan) operating at an accelerating voltage of 80 kV. The core sizes of GNRs were measured by averaging at least 100 nanorods in TEM images. The zeta potentials and hydrodynamic sizes of both GNRs were evaluated by a Malvern Zetasizer Nano ZS (Worcestershire, UK). The spectrophotometric analysis was performed on a Shimadzu UV‐2450 spectrophotometer (Tokyo, Japan). The energy dispersive X‐Ray spectrum of C‐GNRs was measured using STEM described earlier.

*Erythrocytic Hemolysis*: Blood samples were freshly collected from six‐week‐old male C57BL/6 mice (Vital River Laboratory Animal Technology Co. Ltd. China) and added with 10% sodium citrate to avoid coagulation. The erythrocytes were prepared by centrifuging (800 g, 5 min) the blood and PBS washes (five times). The hematocrit of the erythrocyte suspension was controlled at 5% in PBS.

Both PBS‐diluted whole blood (10%) and erythrocyte suspension (about 8 × 10^8^ erythrocytes per mL) were submitted to hemolytic experiments, and the treatments included PBS (negative control, NC), ultrapure water (positive control, PC), and C‐ or P‐GNRs (1.2 × 10^−10^, 2.4 × 10^−10^, 3.6 × 10^−10^, 4.8 × 10^−10^, 6 × 10^−10^, 7.2 × 10^−10^, 8.4 × 10^−10^, and 9.6 × 10^−10^ m). The incubation time was 0.2 h. Then, the samples were centrifuged, and a photograph was taken for visual comparison. The absorbance at 541 nm was measured for the supernatant from each treatment using a Shimadzu UV‐2450 spectrophotometer (Tokyo, Japan). The percentage of hemolysis was expressed as

$$\mathrm{Hemolysis}\;\;=\;\;\frac{\mathrm{Sample}-\mathrm{NC}}{\mathrm{PC}-\mathrm{NC}}\;\;\times \;\;100\%$$

In regard to time‐course for hemolysis induced by C‐GNRs, time points at 0.2, 0.5, 1, 1.5, 2, and 3 h were monitored after the addition of C‐GNRs at different concentrations (1.2 × 10^−10^, 2.4 × 10^−10^, 3.6 × 10^−10^, 4.8 × 10^−10^, 6 × 10^−10^, 7.2 × 10^−10^, 8.4 × 10^−10^, and 9.6 × 10^−10^ m) in erythrocyte suspensions. The potential influence of GNR‐caused conformational changes in hemoglobin on hemolytic measurement was negligible as erythrocytes released abundant free hemoglobin in hemolysis experiments.

The effect of CTAB on hemolysis of erythrocytes was evaluated by incubating whole blood or erythrocyte suspension with different concentrations of CTAB (2.5 × 10^−7^, 5.0 × 10^−7^, 7.5 × 10^−7^, 1.0 × 10^−6^, 1.3 × 10^−6^, 1.5 × 10^−6^, 1.8 × 10^−6^, and 2.0 × 10^−6^ m) for 0.2 h. Both negative control (PBS) and positive control (H_2_O) were tested meanwhile. The concentrations of CTAB, which were comparable to those attached on GNRs, were determined by measuring Br levels in the corresponding C‐GNR solutions using ICP‐MS (Agilent 8800, USA).

*Cellular Internalization of C‐GNRs*: The whole blood samples (0.5 mL) were incubated with C‐GNRs (2 × 10^−10^ and 8 × 10^−10^ m) for 0.2 h. The mixture was centrifuged at 800 g for 5 min, and the cell pellets were washed with PBS for three times. Cellular internalization of C‐GNRs was evaluated by both TEM observation and ICP‐MS analysis. As for the preparation of TEM samples, cell pellets were fixed with 0.1 m natrium cacodylate (Sigma) for 12 h, followed by three‐time wash with cold PBS and 4 h postfixation with 1% osmium tetroxide. The ultrathin sections were prepared and double stained by lead citrate and uranyl acetate. The cellular internalized C‐GNRs were observed and photographed at different magnifications using TEM at the accelerating voltage of 100 kV (Tecnai Spirit, USA). Regarding the sample pretreatment for ICP‐MS analysis, the cells lysates were used, avoiding the nonspecific attachment of C‐GNRs on cell membrane. More specifically, cell lysate was digested with the mixture of H_2_O_2_/HNO_3_ (v:v, 2:1) and HCl/HNO_3_ (v:v, 3:1) at 95 °C for 1 h. After the dilution with ultrapure water, the concentrations of total Au were quantitatively measured by ICP‐MS and calibrated by cell numbers in different samples.

*Identification of Erythrocytic Protein Bound to C‐GNRs*: The whole blood samples were similarly processed as described in the cellular internalization section except that the concentration of C‐GNRs used here was 9.6 × 10^−10^ m. The exposed cell pellets were lyzed and centrifuged (1000 g, 5 min), and then the supernatant was transferred and further submitted to high‐speed centrifugation (5000 g, 15 min). The small pellets at the bottom of eppendorf tubes were washed with PBS for three times and finally analyzed by SDS‐PAGE (4–20% gradient gel coupled with silver staining, Bio‐Rad).

The target protein band (≈15 kDa) was excised from the gel, washed twice with ultrapure water, de‐stained, and digested with ammonium bicarbonate (1 mL, 25 × 10^−3^ m) in 50% acetonitrile for 10 min. The gel pieces were dehydrated under vacuum until they turned white, and then the samples were incubated with dithiothreitol (10 × 10^−3^ m, DTT, Sigma) at 55 °C for 1 h. The excessive DTT was discarded, and the gel pieces were placed in iodoacetamide (55 × 10^−3^ m, IAM, Sigma) solution for 45 min in the dark. The excess of reagent was discarded, and the gel samples were washed twice with ammonium bicarbonate (25 × 10^−3^ m) for 10 min. The ammonium bicarbonate solution containing the protein samples was transferred and combined. They were subsequently submitted to the de‐staining and dehydration processes. After that, the samples were dissolved in ammonium bicarbonate and trypsin solution (1 µL, 1 mg mL^−1^, 15×) was added. The mixture was incubated at 37 °C overnight to prepare the peptide samples and the digestion was stopped by adding 0.1% formic acid.

The resulting peptide samples (10 µL) were analyzed by LC‐MS/MS using Prominence nano‐2D liquid chromatography (Shimadzu, Tokyo, Japan) coupled with MicroTOF QII mass spectrometry (Bruker Daltonics, Karlsruhe, Germany). The samples were separated on a C18 reverse‐phase capillary column (5 µm, 150A, Eprogen). The gradient mobile phase was controlled, that was, 5% eluent B (100% ACN, 0.1% FA) in A (100% H_2_O, 0.1% FA) was initially hold for 4 min, then linearly increased to 50% B during next 26 min, then elevated to 80% B in subsequent 5 min and hold for 10 min, and finally returned to 5% B in 15 min. The flow rate was controlled at 400 nL min^−1^. Peptide analysis was performed using data‐dependent acquisition of one mass spectroscopy scan (ranging from 50 to 2200 *m/z*) followed by MS/MS scans of three most abundant ions in each mass spectral scan. Mass spectral data obtained from LC‐MS/MS were analyzed by searching the nonredundant protein database using Data Analysis Software (Mascot search engine version 2.3.01) for target protein matching.

*Characterization of C‐GNRs Bioconjugated with Hemoglobin*: The morphology of C‐GNRs bioconjugated with hemoglobin was first observed. The sample solutions were prepared by incubating hemoglobin (1 × 10^−5^ m) and C‐GNRs (5 × 10^−9^ m) at 25 °C for 1 h. The reaction mixture (200 µL) was dispersed onto carbon film coated TEM copper grids and air‐dried overnight. The as‐prepared samples were examined and photographed using a Hitachi S‐5500 field‐emission STEM (Tokyo, Japan) operating at an accelerating voltage of 80 kV.

The hydrodynamic sizes and the zeta‐potentials of C‐GNRs bioconjugated with hemoglobin were measured using a Malvern Zetasizer Nano ZS (Worcestershire, UK). Briefly, the solution of C‐GNRs was added with a gradient series of hemoglobin (0, 2 × 10^−7^, 4 × 10^−7^, 1 × 10^−6^, 2 × 10^−6^, 4 × 10^−6^, 1 × 10^−5^, and 2 × 10^−5^ m). After 1 h incubation, the samples were submitted to the corresponding measurements. Totally 20 measurements were taken for each sample. All data points were the average values from three independent runs.

The binding of hemoglobin on C‐GNRs was evaluated by measuring free protein concentration in C‐GNRs and hemoglobin co‐incubation system. Namely, different concentrations of C‐GNRs (2 × 10^−9^, 5 × 10^−9^, 8 × 10^−9^, and 1 × 10^−8^ m) were added in hemoglobin solution (5 × 10^−7^ m), and the incubation lasted for 1 h. The samples were centrifuged (6000 g, 20 min), and the supernatants were submitted to protein quantification using Micro BCA Protein Assay Kit (Thermo Scientific, Rockford, USA) and SDS‐PAGE analysis. The efficiencies of hemoglobin bound on C‐GNRs were calculated by the differential values of protein related to the corresponding C‐GNR amounts.

*Spectroscopic Analysis of Conformational Changes in Hemoglobin*: Hemoglobin samples (1 × 10^−6^ m) were treated with C‐GNRs (0, 1.7 × 10^−10^, 3.4 × 10^−10^, 5.1 × 10^−10^, 6.8 × 10^−10^, and 8.5 × 10^−10^ m) for 0.5 h, and their fluorescence intensities were recorded using a Hitachi F‐4500 fluorescence spectrophotometer (Tokyo, Japan) with the excitation wavelength of 278 nm at 25 °C. The excitation and emission slits were set at 5 nm, and the emission spectrum was recorded from 290 to 410 nm. The fluorescence intensities were corrected for inner filter effects and plotted as a Stern–Volmer plot of *F*_0_/*F* versus C‐GNRs concentration, where *F*_0_ and *F* were fluorescence intensities of the protein with and without C‐GNRs, respectively.

The samples for UV–vis spectra analysis were prepared by incubating hemoglobin (5 × 10^−7^ m) with different concentrations of C‐GNRs (0, 8.5 × 10^−11^, 1.7 × 10^−10^, 2.6 × 10^−10^, 3.4 × 10^−10^, and 4.3 × 10^−10^ m) for 0.5 h. The samples were recorded on a Shimadzu UV‐2450 spectrophotometer (Tokyo, Japan) by scanning the range of 230–600 nm at room temperature. The UV–vis spectrum profiles of hemoglobin upon C‐GNR bioconjugation were depicted by subtracting the absorbance of the corresponding C‐GNR reference solutions (25 °C). Different incubation durations, including 0.5, 1, 2, 4, and 8 h, were also tested for spectrophotometric profile of hemoglobin (the concentrations of the protein and C‐GNRs were controlled at 5 × 10^−7^ m and 4.3 × 10^−10^ m, respectively).

As for CD spectra analysis, the samples were made by treating hemoglobin (2 × 10^−6^ m) with a series concentrations of C‐GNRs (0, 1.7 × 10^−10^, 3.4 × 10^−10^, 5.1 × 10^−10^, and 6.8 × 10^−10^ m) for 0.5 h. CD spectra of hemoglobin in the presence and absence of GNRs were recorded on a JASCO‐815 spectropolarimeter (Tokyo, Japan) with constant nitrogen flush. A quartz cuvette with light path of 1.0 cm was used for measurements with a scan speed of 100 nm min^−1^ and 0.5 nm intervals. CD data at 25 °C in wavelength range of 200–300 nm were collected, and the analysis was performed based on three raw scans. Secondary structural changes in hemoglobin upon the binding with C‐GNRs were analyzed using CDpro software.

*ITC Analysis for Hemoglobin Interaction with C‐GNRs*: The thermodynamic parameters for hemoglobin interaction with C‐GNRs were analyzed by a MicroCal iTC200 isothermal titration calorimeter (Northampton, USA). Hemoglobin solution (20 µL, 5 × 10^−7^ m) was loaded into the syringe and successively injected into the sample cell containing 300 µL of GNRs solution (3.8 × 10^−11^ m in PBS, pH = 7.5) with an interval of 120 s. The dilution heat of the buffer solution was measured and subtracted from the integrated data before curve fitting. The titration data were deconvoluted based on a binding model by a nonlinear least‐squares algorithm using the Origin software package supplied by MicroCal. The number of binding sites on C‐GNRs (*n*), binding constants (*K*_a_), enthalpy change (Δ*H*°), and entropy change (Δ*S*°) were accordingly determined for the evaluation of the binding process between hemoglobin and C‐CTABs.

*Oxygen Carrying and Releasing Tests*: The erythrocytic hemoglobin sample was diluted with sterile isotonic PBS and quantitatively analyzed by BCA assay. Hemoglobin (1.4 × 10^−6^ m) was incubated with C‐GNRs (0, 1.4 × 10^−10^, 2.8 × 10^−10^ m) for 0.5 h. Oxygen binding and releasing were realized by purging oxygen and nitrogen into the solutions described earlier for 5 min at 37 °C, respectively. The absorbance at 505 and 575 nm were immediately recorded for oxyhemoglobin and deoxyhemoglobin on a Shimadzu UV‐2450 spectrophotometer. The oxygen saturation in each sample was calculated according to the method reported by Tsao et al.^40^ The effect of pH ranging from 5.5 to 8.5 in the incubation system was also evaluated for oxygen release of hemoglobin.

All CV measurements were performed at room temperature using a CH 660 electrochemical workstation (CH Instruments, Austin, USA). Three electrodes, including a glassy carbon electrode (GC, 3 mm in diameter, CH Instruments) as the working electrode, a coiled Pt wire as the counter electrode, and a saturated calomel electrode (in saturated KCl) as the reference electrode, were used. The tested solutions were C‐GNRs (2.8 × 10^−10^ m), hemoglobin (2 × 10^−6^ m), and C‐GNRs bioconjugated with hemoglobin at equal concentrations. To minimize dissolved oxygen, they were purged with nitrogen for 15 min. After being loaded onto the surface of GC electrodes, the samples were allowed to evaporate the solvent at 4 °C before measurements.

*Assays for Heme Release of Hemoglobin*: The determination of heme release from hemoglobin upon the interaction with C‐GNRs followed the method reported by Rael et al.^36^ Briefly, hemoglobin (4 × 10^−4^ m) was incubated with PBS (negative control), urea (0.25 m, positive control), and C‐GNRs (1 × 10^−7^ and 1 × 10^−6^ m) for 0.5 h. After converting free hemin to hematin by 1 mL of 0.1 m ammonium acetate (pH 4.0), the samples were washed with chloroform:methanol (5 mL; v:v, 2:1) twice. The combined organic phase was evaporated to dryness by nitrogen, and the residue was re‐dissolved in DMSO:chloroform (0.5 mL; v:v, 1:4). Hematin was then back‐extracted into NaOH (1 mL, 0.1 N) and submitted to spectrophotometric measurement (385 nm). The heme release ratios for C‐GNR exposure groups were calculated by defining negative and positive controls as 0% and 100%, respectively. The effect of CTAB on heme release from hemoglobin was studied by incubating hemoglobin with CTAB (2.1 × 10^−4^ and 2.1 × 10^−3^ m).

The samples as prepared earlier were also submitted to the analysis by a Bruker Daltonics Autoflex III Smartbean MALDI‐TOF mass spectrometer equipped with a 337 nm nitrogen laser at a frequency of 100 Hz (Karlsruhe, Germany), and the data were processed by FlexControl software. The fragment of *m/z* 616.3 was used for the evaluation of heme release.

*Statistical Analysis*: The data in present work were presented as the mean ± SD (standard deviation) of three independent determinations or more. Student's *t*‐test was used for the statistical analysis, and significant differences were evaluated by *p < 0.05 and **p < 0.01, respectively.

## Conflict of Interest

The authors declare no conflict of interest.

## Supporting information

Supplementary
