# Supplementary material for: Hematological Effects of Gold Nanorods on Erythrocytes: Hemolysis and Hemoglobin Conformational and Functional Changes
Source: Adv Sci (Weinh). 2017 Sep 25;4(12):1700296. doi: 10.1002/advs.201700296 (PMC5737108; doi:10.1002/advs.201700296)
Supplement: Supplementary file 1 — Supplementary [file ADVS-4-na-s001.pdf]

Copyright WILEY-VCH Verlag GmbH & Co. KGaA, 69469 Weinheim, Germany, 2017.

ADVANCED SCIENCE
Open Access

Supporting Information

for *Adv. Sci.,* DOI: 10.1002/advs.201700296

Hematological Effects of Gold Nanorods on Erythrocytes: Hemolysis and Hemoglobin Conformational and Functional Changes

Xingchen Zhao, Dawei Lu, S.Qian Liu, Yiling Li, Rui Feng, Fang Hao, Guangbo Qu, Qunfang Zhou, and Guibin Jiang*

WILEY-VCH

Copyright WILEY-VCH Verlag GmbH & Co. KGaA, 69469 Weinheim, Germany, 2017.

Supporting Information

Hematological Effects of Gold Nanorods on Erythrocytes: Hemolysis and Hemoglobin Conformational and Functional Changes

Xingchen Zhao[1,2], Dawei Lu[1,2], Qian Liu[1,2], Yiling Li[1,2], Rui Feng[1,2], Fang Hao[1,2], Guangbo Qu[1,2], Qunfang Zhou[1,2] and Guibin Jiang[1,2]*

[1]State Key Laboratory of Environmental Chemistry and Ecotoxicology, Research Center for Eco-Environmental Sciences, Chinese Academy of Sciences, Beijing 100085, P.R.China

[2] College of Resources and Environment, University of Chinese Academy of Sciences, Beijing, 100049, P.R.China

* Corresponding author:

Tel./fax: +86-10-62849334

E-mail: zhouqf@rcees.ac.cn (Q. Zhou)

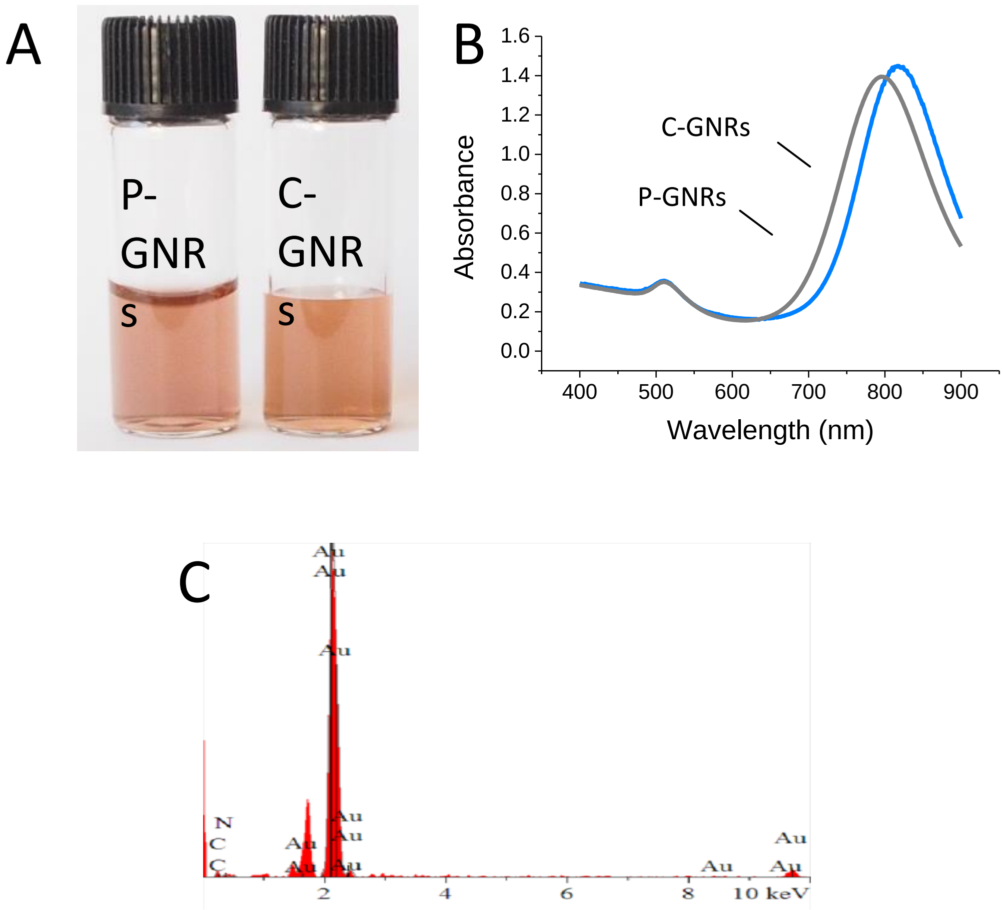

Figure S1. The photograph (A) and the extinction spectrum (B) of as-prepared P- and C-GNR solutions. Absorption peaks at ~511 and ~800 nm show good anisotropy. (C) EDX spectrum of C-GNRs. P-GNRs were derived from the modification of C-GNRs.

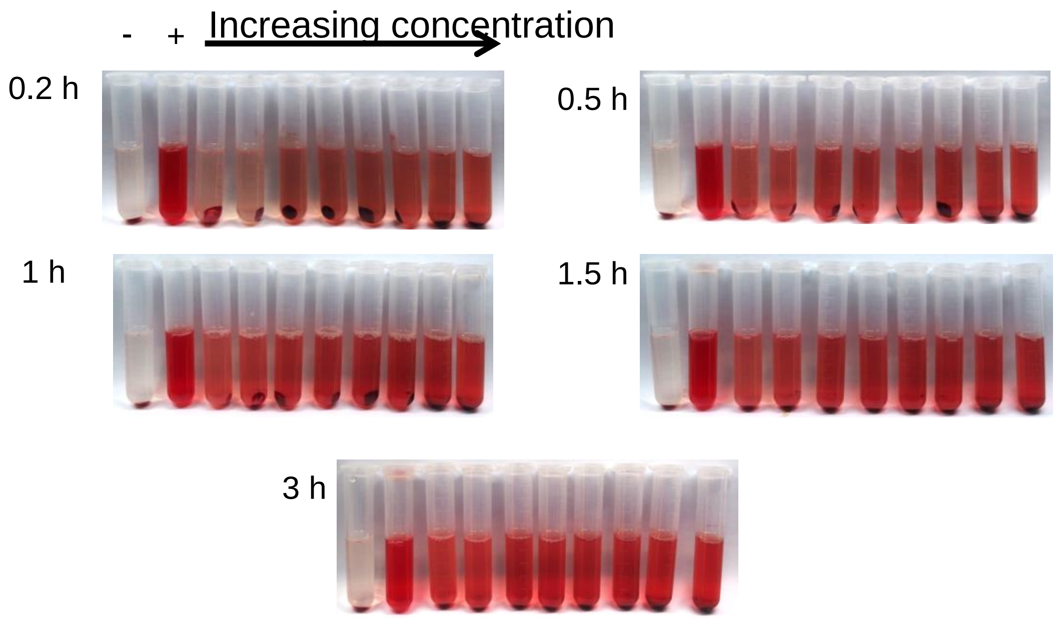

Figure S2. Hemolysis of the erythrocyte suspensions incubated with different concentrations of C-GNRs for 0.2, 0.5, 1, 1.5 and 3 h, respectively. In each panel, the erythrocyte conditions from left to right were negative control (PBS), positive control (H_2O), 1.2×10^{-10}, 2.4×10^{-10}, 3.6×10^{-10}, 4.8×10^{-10}, 6×10^{-10}, 7.2×10^{-10}, 8.4×10^{-10}, and 9.6×10^{-10} M C-GNRs.

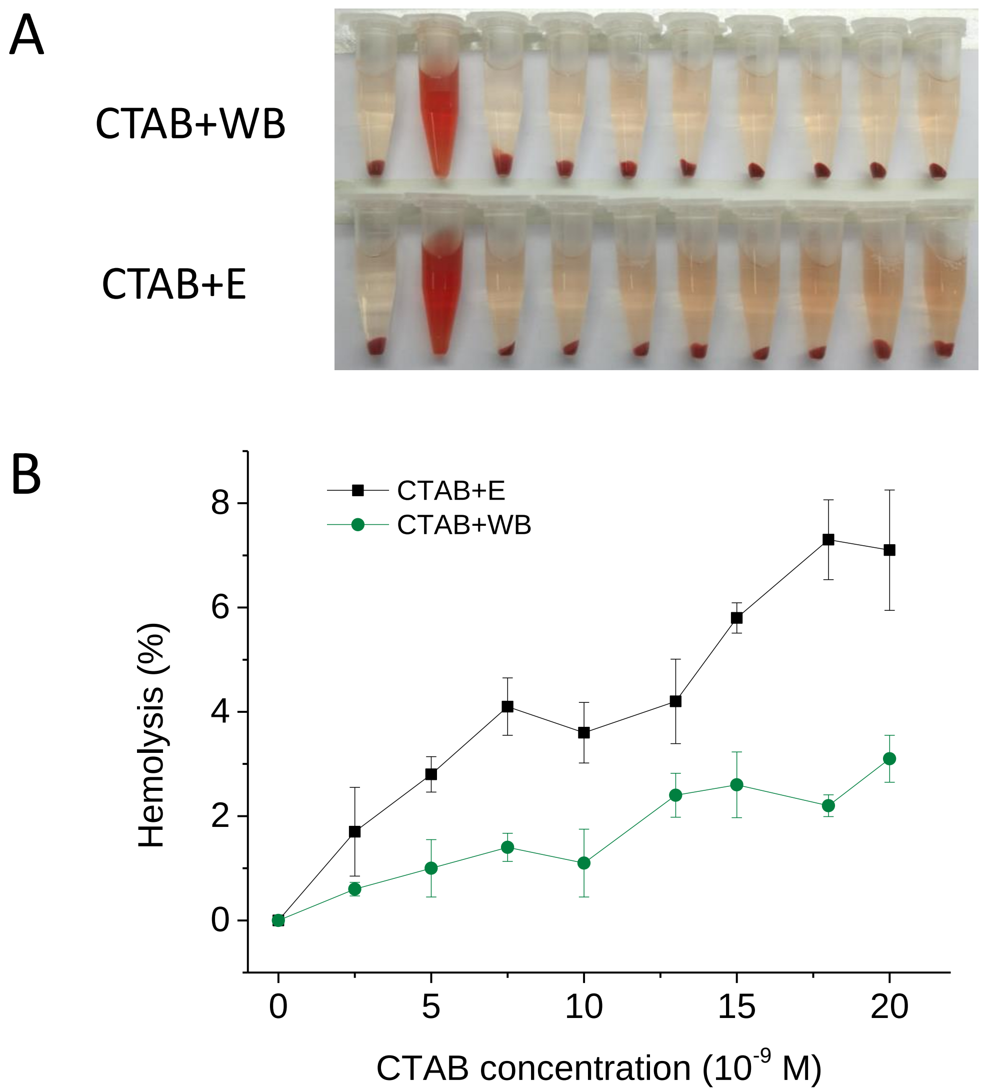

Figure S3. The effect of CTAB on hemolysis of erythrocytes. (A) Photos of the hemolytic effects caused by CTAB after 0.2 h incubation. WB is whole blood, and E is erythrocyte dispersion. The test groups from left to right are PBS (negative control), water (positive control), 2.5×10^{-9}, 5.0×10^{-9}, 7.5×10^{-9}, 1.0×10^{-8}, 1.3×10^{-8}, 1.5×10^{-8}, 1.8×10^{-8}, and 2.0×10^{-8} M CTAB, respectively. The incubation time was 0.2 h. (B) The quantitative analysis for concentration-dependent hemolysis induced by CTAB in whole blood and erythrocyte dispersion based on the absorbance of each sample at λ_{541nm}.

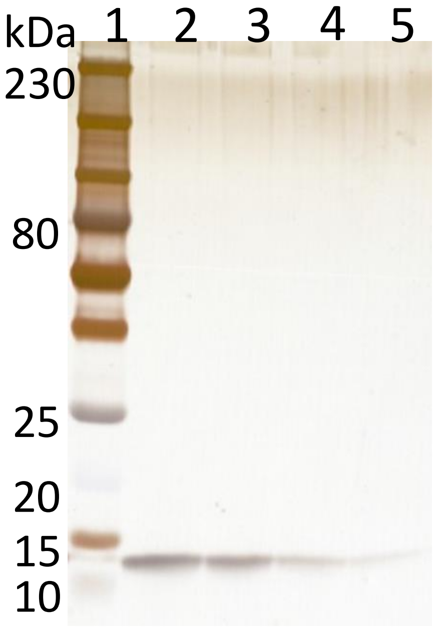

Figure S4. SDS-PAGE analysis for hemoglobin contents in supernatant samples from the incubation systems of C-GNRs and hemoglobin. The initial concentration of hemoglobin was controlled at 5×10^{-7} M, and the concentrations of C-GNRs were 2×10^{-9}, 5×10^{-9}, 8×10^{-9}, and 1×10^{-8} M for Lane 2, 3, 4 and 5, respectively.

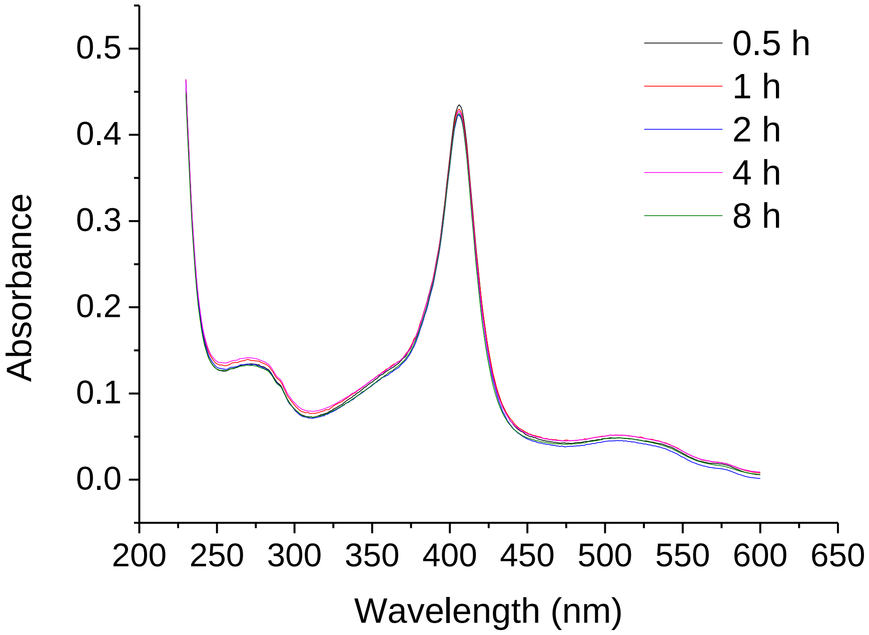

Figure S5. UV-vis spectra of hemoglobin (5×10^{-7} M) incubated with C-GNRs (4.3×10^{-10} M) for different durations.

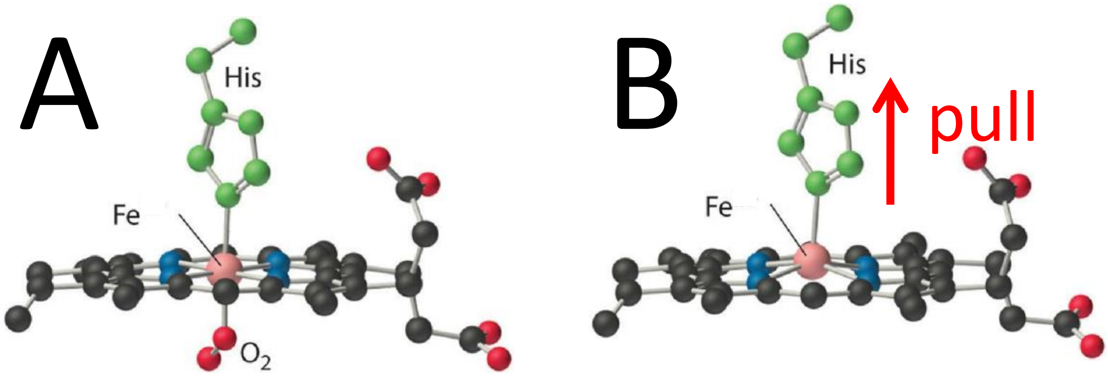

Figure S6. The diagrammatic sketch of oxygen binding (A), releasing (B) modes for hemoglobin. The iron ion moved into the plane of heme when oxygen was bound with hemoglobin. The iron ion was pulled off the plane by the proximal histidine when oxygen molecular was released.

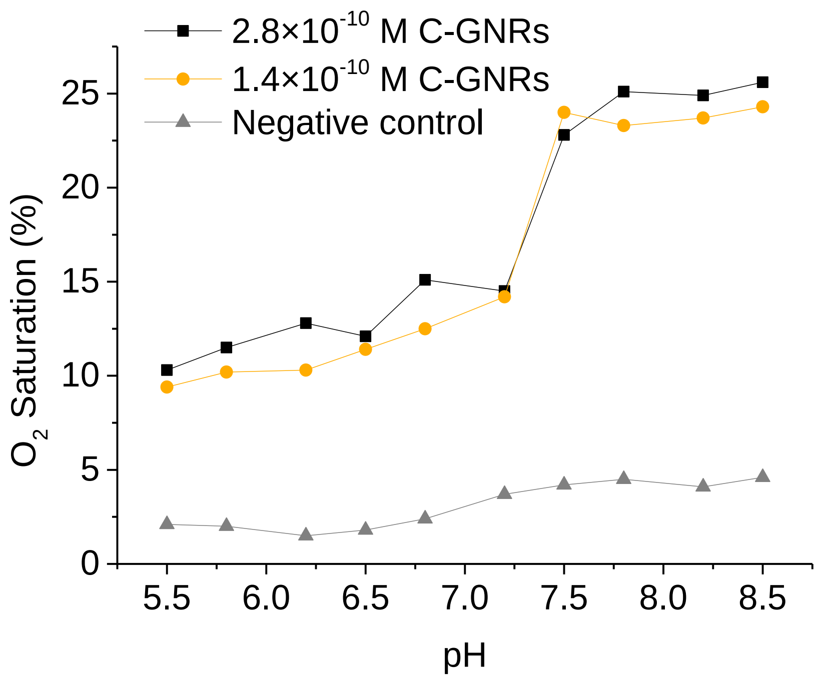

Figure S7. The effect of pH on oxygen release of hemoglobin (2×10^{-6} M) upon C-GNR treatments.

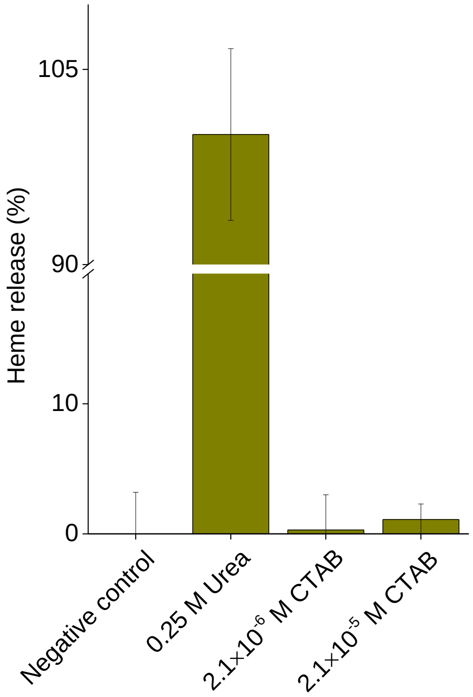

Figure S8. Heme release from hemoglobin caused by CTAB. The concentrations of CTAB (2.1×10^{-6} and 2.1×10^{-5} M) are equal to those in C-GNR solutions (1×10^{-7} and 1×10^{-6} M), and the concentration of hemoglobin is 4×10^{-4} M.
